# Short‐Term Recovery Trajectories of Acute Flares in Knee Pain: A UK‐Netherlands Multicenter Prospective Cohort Analysis

**DOI:** 10.1002/acr.24088

**Published:** 2020-11-27

**Authors:** Martin J. Thomas, Dahai Yu, Elaine Nicholls, Sita Bierma‐Zeinstra, Philip G. Conaghan, Karen J. Stoner, Tuhina Neogi, Emma L. Parry, George Peat

**Affiliations:** ^1^ Keele University Haywood Academic Rheumatology Centre Midlands Partnership NHS Foundation Trust, and Haywood Hospital Burslem Staffordshire UK; ^2^ Keele University Staffordshire UK; ^3^ Erasmus MC–University Medical Centre Rotterdam The Netherlands; ^4^ University of Leeds and NIHR Leeds Biomedical Research Centre Leeds UK; ^5^ Infirst Healthcare Ltd London UK; ^6^ Boston University School of Medicine Boston Massachusetts

## Abstract

**Objective:**

To identify distinct recovery trajectories of acute flares of knee pain and associated participant characteristics.

**Methods:**

Data were from the FLARE randomized controlled trial, a multicenter trial in 27 primary care centers in the UK and Netherlands of 3 regimes of oral nonsteroidal antiinflammatory therapy for acute flares of knee pain. Individuals with a history of inflammatory/crystal arthritis, fibromyalgia, and chronic pain syndrome were excluded. Latent class growth analysis was applied to measures of pain intensity repeated over 5 days to identify distinct recovery trajectories. The concurrent courses of interference with activity, stiffness, and swelling for each trajectory group were modelled using generalized estimating equations. Participant age, sex, obesity, and osteoarthritis diagnosis were described for each trajectory group.

**Results:**

A total of 449 participants were included (median age 55 years, 41% female, 35% obese, and 42% diagnosed with osteoarthritis). A 6‐group cubic model was deemed optimal, with trajectories distinguished by rate of pain reduction and absolute level at final measurement. At the extremes were rapid and near‐complete resolution (n = 41, 9%) and persistent, high pain (n = 25, 6%), but most participants showed a reduction and plateau in pain severity within 3–5 days. Within each pain trajectory group, interference with activity, stiffness, and swelling followed the same course as pain. Baseline characteristics did not differ substantially between trajectory groups.

**Conclusion:**

Even under a well‐adhered to regime of oral nonsteroidal antiinflammatory medication, recovery following acute flares of knee pain is heterogeneous. Our observations that favorable trajectories are apparent within 3–5 days can help to inform treatment decision‐making in the patient–health care professional consultation.

## INTRODUCTION

There is increasing recognition that the natural history of osteoarthritis pain can include intermittent episodes of intense pain (1). Focus groups of people with hip or knee osteoarthritis have suggested that in the early stages of the disease these episodes may be relatively predictable and associated with high‐impact activities, but in later stages can become unpredictable and distressing (1). The underlying nature of these episodes, including the role of inflammation, is still poorly understood, and while a common terminology has yet to be agreed on (2), patients often use the terms “flares” or “flare‐ups” to describe these phenomena, and this is the term under which a new Outcome Measures in Rheumatology–Osteoarthritis Research Society International initiative has recently been launched (3).

**SIGNIFICANCE & INNOVATIONS**

- Recovery trajectories following flares of knee pain managed with oral nonsteroidal antiinflammatory medication are heterogeneous and difficult to predict based on participant characteristics.
- The identification of favorable trajectories within 3–5 days can be used to inform treatment decision‐making in the patient–health care professional consultation.

Part of the unpredictability of flares for patients and health care professionals is knowing how long they will last. A single one‐size‐fits‐all answer is unlikely to be adequate. Long‐term studies have demonstrated that there is no single long‐term course for osteoarthritis symptoms (4, 5, 6), and our hypothesis was that this diversity would be true also of the short‐term course of acute flares. Using a unique trial data set that collected daily measurements from participants experiencing a flare in knee pain, we sought to identify distinct short‐term recovery trajectories of knee pain flares to describe the accompanying changes over time in self‐reported function, stiffness, and swelling, and to explore any participant characteristics associated with trajectory groups.

## MATERIALS AND METHODS

#### Study design

The current study used data from a published randomized controlled trial (RCT), the FLARE RCT (7). The trial was a multicenter, randomized, double‐blind, 3‐arm design testing for noninferiority by comparing a novel lipid formulation of ibuprofen 1,200 mg/day with standard ibuprofen soft‐gel capsules of 1,200 mg/day or 2,400 mg/day. Participants had 5 days of treatment, with day 0 as baseline (no medication), and day 1 as the first treatment day. A total of 27 primary care general practices were recruited across the UK and The Netherlands. People with a history of knee pain flares were identified via medical record review and local community advertising. Participants were screened at local study sites to determine eligibility and invited to return within 24 hours if they experienced a knee pain flare for enrollment and randomization. Independent ethics approval was obtained in both countries (UK: Nottingham Research Ethics Committee East Midlands, Northampton; Netherlands: Independent Review Board, Nijmegen). All participants gave written informed consent.

#### Study population

Community‐dwelling adults ages 40–70 years with a history of ≥1 knee pain flare episode in the last 12 months (with or without treatment), who experienced a new knee pain flare with severity ≥5 on a 0–10 numerical rating scale (NRS), and who attended a baseline assessment within 24 hours of onset, were eligible to take part. Individuals were excluded if they had recent serious illness, fracture, a history of serious heart problems or clinically significant cardiovascular disease, inflammatory arthropathies (including gout), fibromyalgia, chronic pain syndrome, current selective serotonin reuptake inhibitor medication, significant injury or surgery to the knee, recent intraarticular corticosteroid injection into the index knee or systematic glucocorticoids, body mass index (BMI) >39 kg/m^2^, and use of any pain medication within 7 days of study baseline.

#### Data collection and outcomes of interest

Data collection included age, sex, BMI, participant self‐reported osteoarthritis status confirmed by physician questioning, oral antiinflammatory regime allocation, baseline and post‐treatment Western Ontario and McMaster Universities Osteoarthritis Index (WOMAC) NRS (8) scores for pain (0–50), stiffness (0–20), and function (0–170), self‐reported number of days since flare started, knee flare response (proportion of flares “fully controlled/under control” by the end of the 5‐day treatment course one), the proportion beginning the second 5‐day course of oral antiinflammatory medication from day 6, and the proportion of 100% adherence with treatment course one. Participant‐reported average daily pain intensity, pain interference with participant‐nominated activity, stiffness after sitting, lying, or resting, and swelling (all on a 0–10 NRS) were also collected prospectively each day to day 5, at the same time of day as the baseline questionnaire was completed.

#### Statistical analyses

Latent class growth analysis (LCGA) was used to model individual pain intensity trajectories over time and was based on the sample of participants with pain intensity data at all time‐points. Pain intensity was analyzed with a censored normal distribution. A 1‐group quadratic model was initially fitted to the data because we hypothesized that the trajectories in this data set would be nonlinear. A search for the optimal quadratic model was conducted by sequentially increasing the number of groups by 1 until model fit no longer improved. We also explored whether the same optimum model would have been concluded if a cubic model had been assumed, and if any group‐specific cubic terms were statistically significant (*P* < 0.05). Model fit statistics included the Akaike information criterion (AIC), the Bayesian information criterion (BIC), and the sample‐size adjusted BIC (ABIC), with lower absolute values of statistics indicating better model fit. Entropy (value 0–1) was used to indicate how well the model predicted class membership, with values >0.8 desirable (9). We also considered that for a model to be optimal, all class sizes should be >5% of the total sample (to minimize the potential for the specific class not to be replicated in another data set) and that class‐specific average posterior probabilities were >0.7 (10, 11).

To check whether a global solution had been reached in the estimation algorithm, models were rerun using 5,000 different starting values to examine whether the same model likelihood was attained irrespective of starting values. If in >2 final‐stage solutions the highest log likelihood was repeated, a global solution was then concluded (12). We also conducted sensitivity analyses to check whether model results were consistent when the data were modeled using growth mixture models (13), i.e., when the variance and covariance of the growth factors was freely estimated, rather than fixed at zero as in LCGA, or when participants with pain‐intensity data for at least 1 time point were included in the analysis. All models were fitted using maximum likelihood estimation, so that when missing data were included in the model, we assumed them to be missing at random.

Trajectory membership was further examined by plotting the derived trajectories based on the number in the smallest trajectory group and generating a random sample of the same number for each of the other 5 derived trajectories. This process was performed to visually judge the extent to which individual trajectories followed the average trajectory for each group.

In each latent group, the marginal estimation of pain interference, with participant‐nominated activity at each time point as the outcome, was analyzed using generalized estimating equations incorporating age, sex, categorical variable for trajectory group, and a cubic term for time as predictors. Predicted mean estimates at each time point were presented with 95% confidence intervals, calculated using robust standard errors. This process was repeated for self‐reported stiffness and swelling outcomes.

Demographics, clinical characteristics, and the proportion of participants reporting their flare as “fully controlled/under control” at day 5 were described by trajectory group. Unadjusted multinomial logistic regression was used to explore baseline predictors of the trajectory group. Adjusted models were not considered because our sample size was too small for such an analysis to be reliable. Finally, we conducted a subgroup analysis in which we repeated the LCGA in those patients with a diagnosis of osteoarthritis. This procedure was to determine whether the findings in the primary analysis could reasonably be generalized to cases diagnosed with osteoarthritis. Data management and analysis were performed using Stata MP software, version 14.1, and Mplus, version 8.1 (14).

## RESULTS

#### Study population included

Of 462 study participants enrolled and randomized between March 2015 and August 2016, 13 cases had missing data at 1 or more follow‐up time points, leaving 449 participants eligible for inclusion in the complete‐case analysis.

#### Trajectories of recovery

A cubic 6‐group model was deemed the optimal solution based on low AIC, BIC, and ABIC, and high entropy and average posterior probabilities (see Supplementary Table 1, available on the *Arthritis Care & Research* website at http://onlinelibrary.wiley.com/doi/10.1002/acr.24088/abstract). Group membership ranged in size from n = 25 (5.6%, group 6) to n = 143 (31.8%, group 4). In all 6 groups, individual participant trajectories showed a similar spread around the mean, supporting the model fit (see Supplementary Figure 1, available at http://onlinelibrary.wiley.com/doi/10.1002/acr.24088/abstract). The cubic 6‐group growth mixture model, with all parameters freely estimated, failed to converge to a global solution. However, constraining the variance around the quadratic and cubic model terms produced a global solution, albeit where the optimal number of classes was inconsistent across different indices (see Supplementary Tables 2and 3 and Supplementary Figure 2, available at http://onlinelibrary.wiley.com/doi/10.1002/acr.24088/abstract). A similar solution appeared optimal when analysis was restricted to participants with a diagnosis of osteoarthritis (see Supplementary Table 4 and Supplementary Figure 3, available at http://onlinelibrary.wiley.com/doi/10.1002/acr.24088/abstract). The results were also consistent between the complete‐case analysis and the analysis incorporating missing data, given that the rate of missing data in the study was low (3%).

**Figure 1:**
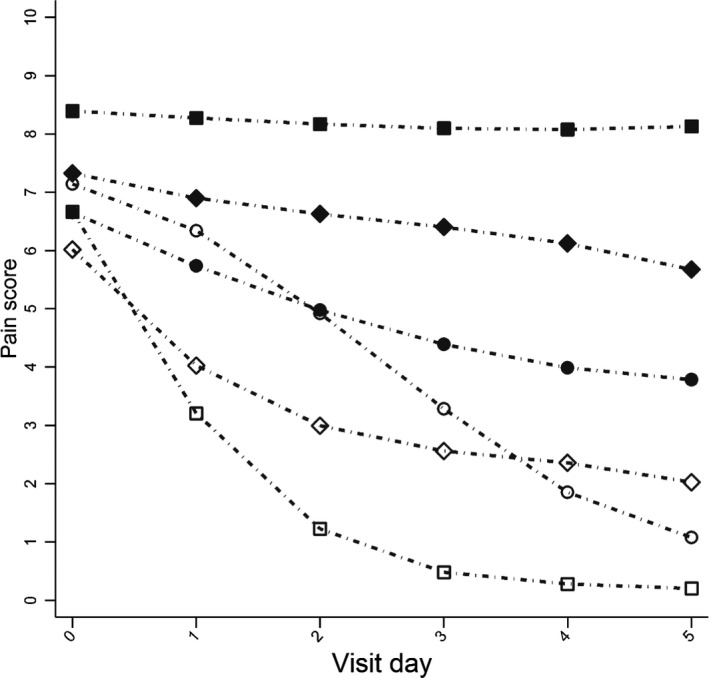
Pain score by group‐based trajectory membership (n = 449). On the x‐axis, 0 = baseline (no medication), and day 1 is the first treatment day. Group 1 (n = 41) □; Group 2 (n = 38) ○; Group 3 (n = 98) ◊; Group 4 (n = 143) ●; Group 5 (n = 104) ♦; Group 6 (n = 25) ■.

Groups were differentiated mainly on the rate of recovery and pain level attained at day 5 (Figure 1).<<F1>> Two groups (group 5 [n = 104] and group 6 [n = 25]) showed modest or minimal reductions in pain, and despite high reported levels of adherence to 5 days of oral nonsteroidal antiinflammatory drugs (NSAIDs), remained at high levels of pain. All other groups showed different rates of pain reduction, with 3 groups experiencing pain scores of <3 on a 0–10 NRS after 5 days of treatment.

Scores for severity of pain interference with participant‐nominated activity and of stiffness closely followed the trajectories in pain severity (see Supplementary Figures 4and 5, available on the *Arthritis Care & Research* website at http://onlinelibrary.wiley.com/doi/10.1002/acr.24088/abstract). Self‐reported severity of swelling also followed a similar course to pain severity, although scores for swelling were systematically lower than for pain severity, particularly at baseline (see Supplementary Figure 6, available e at http://onlinelibrary.wiley.com/doi/10.1002/acr.24088/abstract). Of note, participants who experienced the most rapid and complete resolution of pain (group 1) had much lower self‐reported swelling at baseline than other trajectory groups.

#### Comparison of participant characteristics between trajectory groups

Groups differed on WOMAC subscale scores at baseline and at follow‐up, and this variation was reflected in differences in the proportion who reported their flare as being “fully controlled/under control” at day 5 and opting to begin a second course of oral NSAIDs (Table 1 and see Supplementary Tables 5and 6, available on the *Arthritis Care & Research* website at http://onlinelibrary.wiley.com/doi/10.1002/acr.24088/abstract). Sex, osteoarthritis diagnosis, days since the flare started, and NSAID regime were not strongly associated with group membership. Differences observed between groups for age and BMI did not follow a clear pattern and were not statistically significant (Table 1 and see Supplementary Tables 5and6, available at http://onlinelibrary.wiley.com/doi/10.1002/acr.24088/abstract). While self‐reported adherence with the NSAID regime was generally high in all groups (≥88%), statistically significant between‐group differences were observed, with the lowest adherence seen among the group with the fastest reductions in pain, suggesting discontinuation of NSAIDs due to symptom resolution.

**Table 1:** Description of baseline characteristics across pain trajectory groups*

Characteristic	Overall (n = 449)	Group 1 □ (n = 41)	Group 2 ○ (n = 38)	Group 3 ◊ (n = 98)	Group 4 ● (n = 143)	Group 5 ♦ (n = 104)	Group 6 ■ (n = 25)
Age	55.0 (45.0, 63.0)	55.0 (46.0, 63.0)	58.0 (48.0, 65.0)	48.0 (39.3, 58.3)	59.0 (47.0, 63.3)	56.0 (48.0, 62.0)	49.5 (40.0, 60.3)
Female, no. (%)	185 (41)	19 (46)	16 (42)	34 (35)	59 (41)	45 (43)	12 (48)
Osteoarthritis, no. (%)	187 (42)	16 (39)	16 (42)	41 (42)	64 (45)	38 (37)	12 (48)
Body mass index, kg/m^2^, no. (%)							
<25.0	102 (23)	15 (37)	6 (16)	19 (19)	33 (23)	23 (22)	6 (24)
25.0–29.9	192 (43)	15 (37)	16 (42)	54 (55)	58 (41)	43 (41)	6 (24)
30.0–39.0	155 (35)	11 (27)	16 (42)	25 (26)	52 (36)	38 (37)	13 (52)
Baseline severe pain (≥7 of 10 NRS), no. (%)	279 (62)	23 (56)	34 (90)	32 (33)	82 (57)	84 (81)	24 (96)
Baseline WOMAC score							
Pain (0–50)	29 (22, 34)	24 (19, 28)	32 (28, 35)	23 (19, 28)	28 (23, 33)	33 (27, 37)	40 (35, 42)
Stiffness (0–20)	13 (10, 15)	11 (8, 14)	13 (11, 15)	9 (2, 12)	13 (10, 15)	14 (12, 16)	16 (14, 18)
Function (0–170)	92 (69, 115)	74 (50, 90)	100 (89, 123)	72 (52, 93)	95 (74, 113)	102 (75, 124)	126 (112, 136)
Days since flare started, no. (%)							
0	123 (27)	14 (34)	11 (29)	27 (28)	40 (28)	26 (25)	5 (20)
1	307 (68)	26 (63)	27 (71)	68 (69)	96 (67)	74 (71)	16 (64)
2	12 (3)	1 (2)	0 (0)	2 (2)	4 (3)	3 (3)	2 (8)
≥3	7 (2)	0 (0)	0 (0)	1 (1)	3 (2)	1 (1)	2 (8)
NSAID regime, no. (%)							
Lipid 1,200 mg/day	142 (32)	14 (34)	16 (42)	25 (26)	47 (33)	31 (30)	9 (36)
Soft gel 1,200 mg/day	153 (34)	13 (32)	9 (24)	33 (34)	44 (31)	44 (42)	10 (40)
Soft gel 2,400 mg/day	154 (34)	14 (34)	13 (34)	40 (41)	52 (36)	29 (28)	6 (24)
End of course 1 WOMAC score							
Pain (0–50)	14 (6, 22)	1 (0, 3)	5 (3, 8)	8 (4, 13)	16 (12, 21)	24 (18, 30)	36 (33, 42)
Stiffness (0–20)	6 (3, 10)	0 (0, 0)	3 (2, 4)	4 (2, 5)	7 (5, 10)	10 (8, 12)	16 (14, 18)
Function (0–170)	46 (18, 72)	3 (0, 8)	18 (10, 27)	22 (11, 40)	55 (42, 71)	72 (52, 94)	124 (91, 136)
Flare fully controlled/under control, no. (%)	246 (55)	40 (98)	37 (97)	69 (70)	73 (51)	27 (26)	0 (0)
Beginning second course of NSAIDs , no. (%)	131 (29)	1 (2)	0 (0)	19 (19)	42 (29)	52 (50)	17 (68)
100% adherent with treatment course one, no. (%)	414 (92)	36 (88)	38 (100)	88 (90)	132 (92)	95 (91)	25 (100)

* Values are the median (interquartile range), unless indicated otherwise.. NRS = numerical rating scale; WOMAC = Western Ontario and McMaster Universities Osteoarthritis Index; NSAID = nonsteroidal antiinflammatory drug.

## DISCUSSION

Our findings demonstrate that acute flares of knee pain do not follow a predictable, set course. From pain levels that were initially moderate‐to‐severe, we identified a range of recovery trajectories from rapid and substantial symptom improvement within 3 days to minimal short‐term improvement. Unfortunately we found no strong predictors of recovery trajectory, although data on several potentially relevant determinants of outcome (e.g., occupational exposures, low mood) were not collected. Since all participants in our study received a 5‐day course of oral NSAID preparations of comparable efficacy, and with high self‐reported adherence, we can be more confident that differences in recovery trajectory are unlikely to be explained simply by differences in treatment. Indeed, we should recognize that the natural course of flares under less optimized, real‐world conditions are likely to be less favorable than observed in this study. Furthermore, with the absence of a no‐treatment control for comparison, whether the same patterns and frequency of patterns would occur under other or no treatment conditions cannot be known.

The age of participants in our study may be important. Studies of long‐term symptom trajectories in knee pain have recruited participants with a mean age ranging from 56 to 71 years. The age of participants in the current study was at the lower end of this range (median age 55 years) and comparable with that of the Cohort Hip and Cohort Knee cohort of early osteoarthritis (e.g., 4). Following the exclusion of potential participants with a history of inflammatory/crystal arthropathy, fibromyalgia, and chronic pain syndrome, our study findings most likely reflect acute flares in relatively early knee osteoarthritis. Thus, our observations provide some empirical support for the qualitative finding of Hawker et al that flares may be present in an early phase of osteoarthritis (1).

In this study, acute flares were self‐declared by participants, a pragmatic decision in the absence of more robust criteria, and one with some face and construct validity. Nevertheless, the flares are still likely to represent heterogeneous underlying pathophysiologic processes. Future studies using imaging to assess the role of joint inflammation, for example, may be insightful. The resolution of pain appeared to track the resolution of self‐reported swelling, and those participants with the most rapid pain recovery had the least swelling at baseline, consistent with the proposal of Marty et al (15) that knee effusion is an important (but not essential) component of knee osteoarthritis flares. We acknowledge that the 5‐day study period was short, and future studies with longer follow‐up could more accurately establish both the time to resolution of flares (particularly for those groups whose pain did not resolve over 5 days) and the frequency and interval of recurrence.

What are the implications of this research? We provide some evidence that could inform the conversation between health care professionals and patients about the usual expected course of a flare but also when an unfavorable trajectory might become apparent. Our study does not provide evidence on how these should be managed. However, we note that if achieving rapid and substantial symptom improvement in all patients is unrealistic, an alternative is to attempt to shift patients’ flares into an adjacent, more favorable trajectory. This more modest goal could still produce important reductions in disability days and time spent in moderate‐to‐severe pain.

## AUTHOR CONTRIBUTIONS

All authors were involved in drafting the article or revising it critically for important intellectual content, and all authors approved the final version to be submitted for publication. Dr. Thomas had full access to all of the data in the study and takes responsibility for the integrity of the data and the accuracy of the data analysis.

### Study conception and design

Thomas, Yu, Conaghan, Stoner, Peat.

### Acquisition of data

Bierma‐Zeinstra.

### Analysis and interpretation of data

Thomas, Yu, Nicholls, Conaghan, Stoner, Neogi, Parry, Peat.

## Supporting information

Supplementary Material
