# Supplementary material for: Short‐Term Recovery Trajectories of Acute Flares in Knee Pain: A UK‐Netherlands Multicenter Prospective Cohort Analysis
Source: Arthritis Care Res (Hoboken). 2020 Nov 27;72(12):1687–92. doi: 10.1002/acr.24088 (PMC7145729; doi:10.1002/acr.24088)
Supplement: Supplementary file 1 — Supplementary Material [file ACR-72-1687-s001.docx]

**Supplementary Data**

**Tables**

**S1** Goodness-of-fit statistics for quadratic and cubic models for pain trajectory, all participants (n=449)

**S2** Model constraints applied to the 6-group GMM model

**S3** Pain scale: goodness-of-fit statistics for a cubic GMM model with the variance of the cubic and quadratic term fixed at 0

**S4** Goodness-of-fit statistics for quadratic and cubic models for pain trajectory, diagnosed osteoarthritis only (n=187)

**S5** Differences in baseline characteristics by pain trajectory group, expressed relative risk ratios from multinomial logistic regression, all participants

**S6** Differences in outcomes by trajectory group, expressed as relative risk ratios from multinomial logistic regression, all participants

**Figures**

**S1** Plots of estimated means of the final model and the observed individual trajectories, by pain trajectory group, all participants

**S2** Trajectory plots of the constrained GMM model with varying numbers of groups (i.e., with the variance of the cubic and quadratic terms constrained at 0)

**S3** Pain score by group-based trajectory membership, diagnosed osteoarthritis only (n=187)

**S4** Pain interference with participant-nominated activity (◼), by pain (⭘) trajectory group, all participants

**S5** Stiffness (◼), by pain (⭘) trajectory group, all participants

**S6** Swelling (◼), by pain (⭘) trajectory group, all participants

**Supplementary Table 1** Goodness-of-fit statistics for quadratic and cubic models for pain trajectory, all participants (n=449)

| Model | No of class | AIC | BIC | ABIC | Entropy | L | Sample size in each class, n (%) | Average posterior probability |
| --- | --- | --- | --- | --- | --- | --- | --- | --- |
| Quadratic | 1 | -5853.85 | -5862.70 | -5860.01 |  | -5850.85 | 449 (100) | 1 |
|  | 2 | -5419.54 | -5440.18 | -5433.91 | .8055834 | -5412.54 | 160 (35.6), 289 (64.4) | 0.91, 0.96 |
|  | 3 | -5317.45 | -5349.89 | -5340.04 | .7762766 | -5306.45 | 56 (12.5), 220 (49.0), 173 (38.5) | 0.91, 0.88, 0.89 |
|  | 4 | -5300.87 | -5345.11 | -5331.67 | .7777202 | -5285.87 | 22 (4.9), 62 (13.8), 226 (50.3), 139 (31.0) | 0.91, 0.83, 0.84, 0.90 |
|  | 5 | -5302.39 | -5383.43 | -5341.41 | .9961464 | -5283.39 | 2 (0.5), 23 (5.1), 60 (13.4), 226 (50.3), 138 (30.7) | 0.91, 0.87, 0.84, 0.84, 0.90 |
|  | 6 | -5305.11 | -5372.95 | -5352.34 | .7973556 | -5282.11 | 151 (33.6), 207 (46.1), 22 (4.9), 60 (13.4), 3 (0.7), 6 (1.3) | 0.82, 0.83, 0.91, 0.79, 0.71, 0.68 |
|  | 7 | -5309.00 | -5388.63 | -5364.44 | .6773106 | -5282.00 | 3 (0.7), 21 (4.7), 2 (0.5), 61 (13.6), 92 (20.5), 125 (27.8), 145 (32.3) | 0.70, 0.92, 0.81, 0.75, 0.50, 0.60, 0.80 |
|  |  |  | | | | | | |
| Cubic | 1 | -5726.36 | -5743.91 | -5739.43 |  | -5724.36 | 449 (100) | 1 |
|  | 2 | -5098.32 | -5128.30 | -5129.34 | .8780987 | -5098.80 | 244 (54.3), 205 (45.7) | 0.97, 0.96 |
|  | 3 | -4809.48 | -4853.72 | -4840.28 | .9210879 | -4794.48 | 88 (19.6), 231 (51.5), 130 (29.0) | 0.97, 0.97, 0.97 |
|  | 4 | -4716.28 | -4745.20 | -4757.28 | .8697848 | -4696.21 | 73 (16.3), 178 (39.6), 135 (30.1), 63 (14.0) | 0.95, 0.92, 0.90, 0.94 |
|  | 5 | -4652.44 | -4726.17 | -4703.77 | .9510735 | -4627.44 | 28 (6.2), 83 (18.5), 194 (43.2), 114 (25.4), 30 (6.7) | 0.96, 0.93, 0.91, 0.92, 0.92 |
|  | **6** | **-4591.59** | **-4680.07** | **-4653.19** | **.993965** | **-4561.59** | **38 (8.5), 98 (21.8), 41 (9.1), 143 (31.9), 104 (23.2), 25 (5.6)** | **0.99, 0.93, 0.88, 0.88, 0.89, 0.96** |
|  | 7 | -4548.66 | -4651.89 | -4620.53 | .9472097 | -4513.66 | 75 (16.7), 26 (5.8), 120 (26.7), 58 (12.9), 55 (12.3), 94 (20.9), 21 (4.7) | 0.86, 0.96, 0.85, 0.93, 0.83, 0.92, 0.95 |

AIC, Akaike Information Criteria; BIC, Bayesian Information Criteria; ABIC, Sample-size adjusted BIC; L, Log likelihood.

**Supplementary Table 2** Model constraints applied to the 6-group cubic GMM model

| Model | Constraint | | | Outcome |
| --- | --- | --- | --- | --- |
|  | Residual variances to be constrained to be equal across trajectory classes and time-points | Variance around the cubic term constrained to be equal to 0 | Variance around the quadratic term constrained to be equal to 0 |  |
| GMM1 | No | No | No | Best log likelihood not replicated |
| GMM2 | Yes | No | No | Best log likelihood not replicated |
| GMM3 | No | Yes | No | Best log likelihood not replicated |
| GMM4 | Yes | Yes | No | Model could not be fully estimated under the model constraints listed |
| GMM5 | No | Yes | Yes | Best log likelihood replicated |

Footnote: The GMM models are fitted using Mplus version 8.1. GMM, Growth Mixture Model.

**Supplementary Table 3** Pain scale: goodness-of-fit statistics for a cubic GMM model with the variance of the cubic and quadratic term fixed at 0

| No of groups | AIC | BIC | ABIC | Entropy | L | Sample size in each class, n (%) | Average posterior probability |
| --- | --- | --- | --- | --- | --- | --- | --- |
| 1 | 9182 | 9235 | 9194 |  | -4578 | 449 (100) | 1.00 |
| 2 | 8853 | 8927 | 8870 | 0.80 | -4409 | 131 (29), 318 (71) | 0.91, 0.96 |
| 3 | 8830 | 8924 | 8851 | 0.81 | -4392 | 299 (67), 130 (29), 20 (4) | 0.94, 0.86, 0.83 |
| 4 | 8802 | 8917 | 8828 | 0.80 | -4373 | 123 (27), 275 (61), 32 (7), 19 (4) | 0.88, 0.90, 0.76, 0.87 |
| 5 | 8790 | 8926 | 8821 | 0.76 | -4362 | 31 (7), 19 (4), 65 (14), 53 (12), 281 (63) | 0.75, 0.82, 0.77, 0.75, 0.90 |
| 6 | 8776 | 8932 | 8811 | 0.67 | -4350 | 172 (38), 60 (13), 33 (7), 56 (12), 17 (4), 111 (25) | 0.76, 0.77, 0.75, 0.73, 0.81, 0.78 |
| 7 | 8771 | 8948 | 8811 | 0.71 | -4343 | 15 (3), 2 (0), 97 (22), 51 (11), 65 (14), 181 (40), 38 (8) | 0.83, 0.99, 0.77, 0.74, 0.78, 0.77, 0.74 |

Footnote: The GMM models are fitted using Mplus version 8.1. AIC, Akaike Information Criteria; BIC, Bayesian Information Criteria; ABIC, Sample-size adjusted BIC; L, Log likelihood.

**Supplementary Table 4** Goodness-of-fit statistics for quadratic and cubic models for pain trajectory, diagnosed osteoarthritis only (n=187)

| Model | No of class | AIC | BIC | ABIC | Entropy | L | Sample size in each class, n (%) | Average posterior probability |
| --- | --- | --- | --- | --- | --- | --- | --- | --- |
| Quadratic | 1 | -2421.69 | -2426.54 | -2429.23 |  | -2418.69 | 187 (100) | 1 |
|  | 2 | -2251.89 | -2263.20 | -2269.47 | 0.8980591 | -2244.89 | 85 (45.5), 102 (54.5) | 0.92, 0.92 |
|  | 3 | -2203.10 | -2220.87 | -2230.72 | 0.9228409 | -2192.10 | 24 (12.8), 113 (60.4), 50 (26.7) | 0.92, 0.92, 0.92 |
|  | 4 | -2198.08 | -2222.32 | -2235.75 | 0.9313741 | -2183.08 | 6 (3.2), 28 (15.0), 104 (55.6), 49 (26.2) | 0.89, 0.83, 0.92, 0.90 |
|  | 5 | -2201.24 | -2231.93 | -2248.95 | 0.8749437 | -2182.24 | 5 (2.7), 46 (24.6), 21 (11.2), 74 (39.6), 41 (21.9) | 0.92, 0.65, 0.80, 0.70, 0.87 |
|  | 6 | -2204.50 | -2241.66 | -2262.26 | 0.8844616 | -2181.50 | 5 (2.7), 47 (25.1), 21 (11.2), 25 (13.4), 87 (46.5), 2 (1.1) | 0.92, 0.81, 0.79, 0.53, 0.73, 0.82 |
|  | 7 | -2208.50 | -2252.12 | -2276.31 | 0.8357559 | -2181.50 | 2 (1.1), 21 (11.2), 68 (36.4), 5 (2.7), 0 (0.0), 42 (22.5), 49 (26.2) | 0.82, 0.79, 0.43, 0.92, 0, 0.49, 0.79 |
|  |  |  | | | | | | |
| Cubic | 1 | -2389.97 | -2398.05 | -2402.53 |  | -2384.97 | 187 (100) | 1 |
|  | 2 | -2114.27 | -2130.43 | -2139.38 | 0.9626631 | -2104.27 | 131 (70.1), 56 (29.9) | 0.97, 0.97 |
|  | 3 | -1964.48 | -1988.71 | -2002.15 | 0.9725973 | -1949.48 | 38 (20.3), 101 (54.0), 48 (25.7) | 0.96, 0.98, 0.97 |
|  | 4 | -1931.48 | -1963.79 | -1981.71 | 0.9976213 | -1911.48 | 8 (4.3), 44 (23.5), 91 (48.7), 44 (23.5) | 0.96, 0.93, 0.95, 0.97 |
|  | 5 | -1929.55 | -1969.94 | -1992.34 | 1 | -1904.55 | 2 (1.1), 6 (3.2), 44 (23.5), 91 (48.7), 44(23.5) | 0.998, 0.99, 0.93, 0.95, 0.97 |
|  | **6** | **-1868.99** | **-1917.45** | **-1944.33** | **1** | **-1838.99** | **3 (1.6), 50 (26.7), 27 (14.4), 59 (31.6), 37 (19.8), 11 (5.9)** | **0.99, 0.89, 0.96, 0.88, 0.95, 0.98** |
|  | 7 | -1873.99 | -1930.53 | -1961.89 | 1 | -1838.99 | 0 (0.0), 3 (1.6), 27 (14.4), 50 (26.7), 59 (31.6), 37 (19.8), 11 (5.9) | 0, 0.99, 0.96, 0.89, 0.88, 0.95, 0.98 |

AIC, Akaike Information Criteria; BIC, Bayesian Information Criteria; ABIC, Sample-size adjusted BIC; L, Log likelihood.

**Supplementary Table 5** Differences in baseline characteristics by pain trajectory group, expressed as relative risk ratios from multinomial logistic regression, all participants

|  | | **Group 1**  **🞏** | **Group 2**  **🞅** | **Group 3**  **◇** | **Group 4**  **●** | **Group 5**  **◆** | **Group 6**  **■** | **Likelihood ratio test** |
| --- | --- | --- | --- | --- | --- | --- | --- | --- |
| N | | 41 | 38 | 98 | 143 | 104 | 25 |  |
| Age | | 0.99 (0.96, 1.02) | 1.01 (0.98, 1.04) | 1.01 (0.99, 1.04) | Reference | 0.98 (0.96, 1.00) | 0.99 (0.96, 1.02) | χ^2^=10.16, p=0.07 |
| Female gender | | 1.23 (0.61, 2.47) | 1.04 (0.50, 2.14) | 0.76 (0.44, 1.29) | Reference | 1.09 (0.65, 1.81) | 1.31 (0.56, 3.08) | χ^2^=2.85, p=0.72 |
| Osteoarthritis | | 0.79 (0.39, 1.60) | 0.90 (0.44, 1.85) | 0.89 (0.53, 1.49) | Reference | 0.71 (0.42, 1.19) | 1.14 (0.49, 2.67) | χ^2^=2.23, p=0.82 |
| Body Mass Index (kg/m^2^) | <25.0 | 1 | 1 | 1 | Reference | 1 | 1 | χ^2^=16.68, p=0.08 |
|  | 25.0-29.9 | 0.57 (0.25, 1.31) | 1.52 (0.54, 4.25) | 1.62 (0.82, 3.18) | Reference | 1.06 (0.55, 2.06) | 0.57 (0.17, 1.91) |  |
|  | 30.0-39.0 | 0.47 (0.19, 1.14) | 1.69 (0.60, 4.76) | 0.84 (0.40, 1.75) | Reference | 1.05 (0.53, 2.06) | 1.38 (0.48, 3.97) |  |
| Baseline severe pain | | 0.95 (0.47, 1.91) | 6.32 (2.13, 18.76) | 0.36 (0.21, 0.62) | Reference | 3.12 (1.73, 5.63) | 17.9 (2.35, 135.6) | χ^2^=84.74, p=<0.001 |
| Baseline WOMAC pain score (0-50) | | 0.93 (0.88, 0.97) | 1.06 (1.01, 1.12) | 0.93 (0.89, 0.96) | Reference | 1.08 (1.05, 1.13) | 1.24 (1.15, 1.34) | χ^2^=120.70, p=<0.001 |
| Baseline WOMAC stiffness score (0-20) | | 0.88 (0.81, 0.96) | 1.04 (0.94, 1.14) | 0.95 (0.89, 1.02) | Reference | 1.07 (1.00, 1.15) | 1.40 (1.19, 1.64) | χ^2^=44.21, p=<0.001 |
| Baseline WOMAC function score (0-170) | | 0.97 (0.96, 0.98) | 1.01 (1.00, 1.02) | 0.97 (0.96, 0.98) | Reference | 1.01 (1.00, 1.02) | 1.05 (1.03, 1.07) | χ^2^=110.33, p=<0.001 |
| Days since flare started: | 0 day | 1 | 1 | 1 | Reference | 1 | 1 | χ^2^=12.34, p=0.65 |
|  | 1 day | 0.77 (0.37, 1.63) | 1.02 (0.46, 2.26) | 1.05 (0.59, 1.87) | Reference | 1.19 (0.66, 2.12) | 1.33 (0.46, 3.89) |  |
|  | 2 days | 0.71 (0.07, 6.94) | - | 0.74 (0.13, 4.33) | Reference | 1.15 (0.24, 5.58) | 4.00 (0.58, 27.7) |  |
|  | ≥ 3 days | - | - | 0.49 (0.05, 5.00) | Reference | 0.51 (0.05, 5.20) | 5.34 (0.71, 40.1) |  |
| NSAID regime: | Lipid 1200 | 1 | 1 | 1 | Reference | 1 | 1 | χ^2^=10.42, p=0.40 |
|  | Soft gel 1200 | 0.99 (0.42, 2.34) | 0.60 (0.24, 1.50) | 1.41 (0.73, 2.74) | Reference | 1.52 (0.82, 2.81) | 1.19 (0.44, 3.19) |  |
|  | Soft gel 2400 | 0.90 (0.39, 2.09) | 0.73 (0.32, 1.69) | 1.45 (0.77, 2.73) | Reference | 0.85 (0.45, 1.61) | 0.60 (0.20, 1.82) |  |
| NSAID, Nonsteroidal anti-inflammatory drug; WOMAC, Western Ontario & McMaster Osteoarthritis Index.  For empty cells with no cases estimates could not be derived. | | | | | | | | |

**Supplementary Table 6** Differences in outcomes by trajectory group, expressed as relative risk ratios from multinomial logistic regression, all participants

|  | Group 1  🞏 | Group 2  🞅 | Group 3  ◇ | Group 4  ● | Group 5  ◆ | Group 6  ■ | **Likelihood ratio test** |
| --- | --- | --- | --- | --- | --- | --- | --- |
| N | 41 | 38 | 98 | 143 | 104 | 25 |  |
| End course 1 WOMAC pain score (0-50) | 0.54 (0.46, 0.63) | 0.73 (0.67, 0.80) | 0.82 (0.78, 0.87) | Reference | 1.10 (1.07, 1.14) | 1.45 (1.30, 1.63) | χ^2^=457.14, p=<0.001 |
| End course 1 WOMAC stiffness score (0-20) | 0.43 (0.34, 0.53) | 0.65 (0.56, 0.75) | 0.74 (0.68, 0.81) | Reference | 1.17 (1.09, 1.25) | 1.92 (1.57, 2.35) | χ^2^=330.99, p=<0.001 |
| End course 1 WOMAC function score (0-170) | 0.84 (0.80, 0.88) | 0.92 (0.90, 0.95) | 0.95 (0.93, 0.96) | Reference | 1.02 (1.01, 1.03) | 1.07 (1.05, 1.09) | χ^2^=387.89, p=<0.001 |
| Flare fully controlled/Under control by the end of course 1 | 38 (5.13, 287) | 36 (4.7, 266) | 2.28 (1.32, 3.93) | Reference | 0.34 (0.19, 0.58) | - | χ^2^=163.34, p=<0.001 |
| Beginning second course of NSAIDs | 0.06 (0.01, 0.45) | - | 0.58 (0.31, 1.07) | Reference | 2.40 (1.42, 4.07) | 5.11 (2.05, 12.8) | χ^2^=87.67, p=<0.001 |
| 100% compliant with treatment course 1 | 0.60 (0.20, 1.84) | - | 0.73 (0.30, 1.80) | Reference | 0.88 (0.35, 2.21) | - | χ^2^=12.01, p=0.03 |
| NSAIDs, Nonsteroidal anti-inflammatory drugs; WOMAC Western Ontario & McMaster Osteoarthritis Index. | | | | | | | |

For empty cells with no cases estimates could not be derived.

**Supplementary Figure 1** Plots of estimated means of the final model and the observed individual trajectories, by pain trajectory group, all participants **
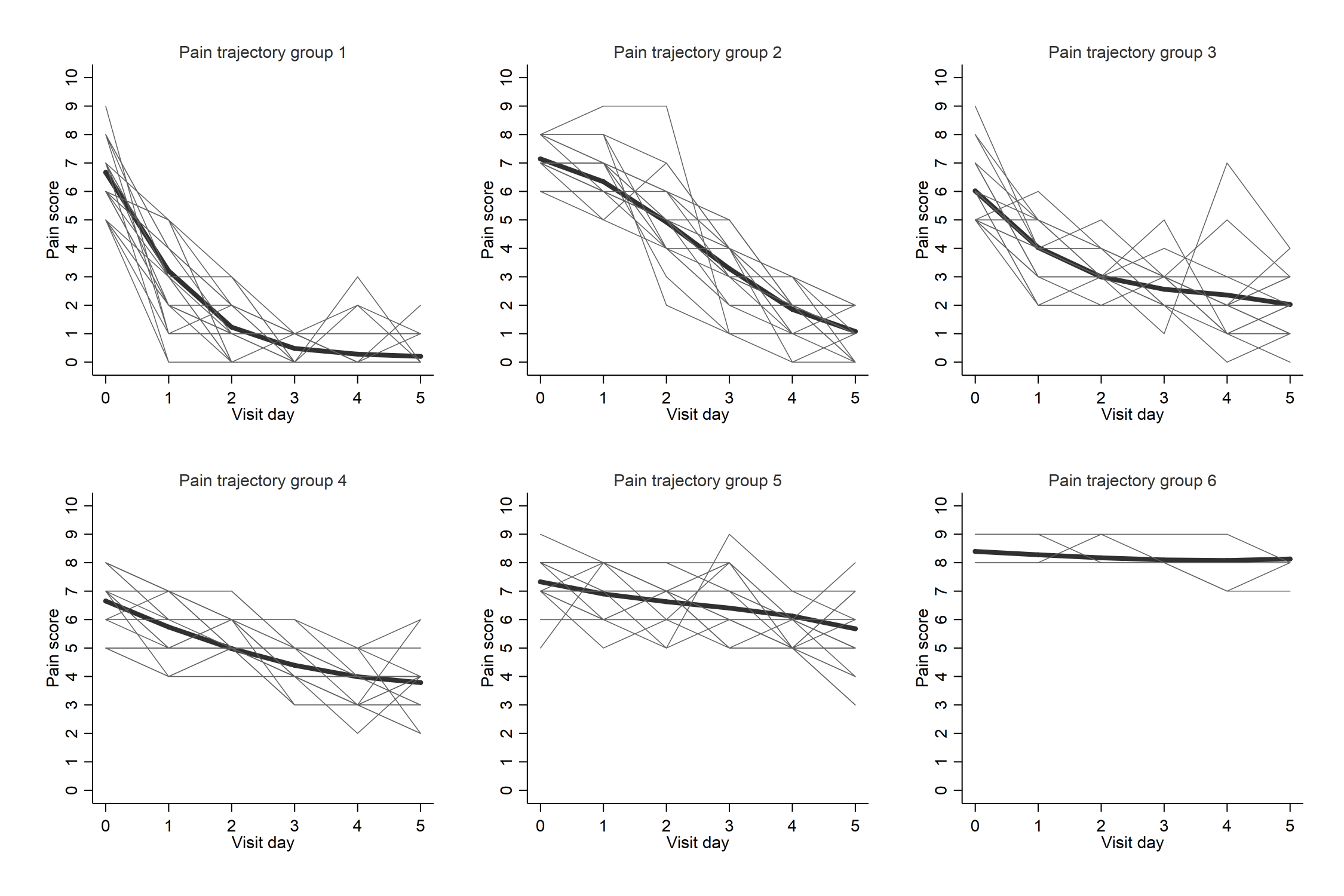
**

**Supplementary Figure 2** Trajectory plots of the constrained GMM model with varying numbers of groups (i.e. with the variance of the cubic and quadratic terms constrained to 0)

**1-group model 2-group model 3-group model 4-group model**

**
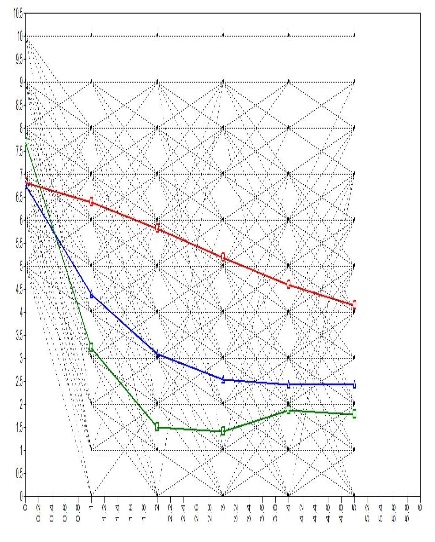

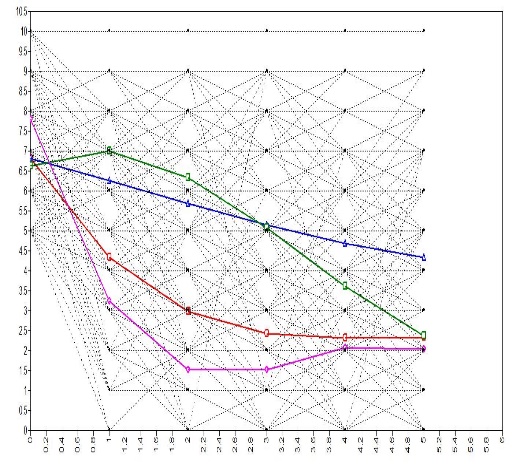

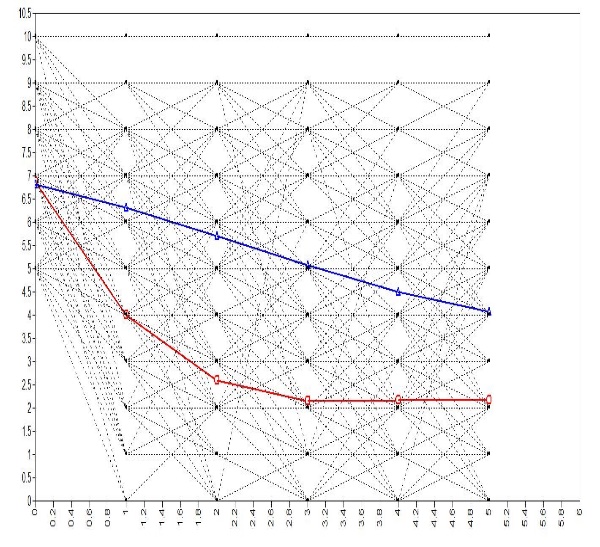

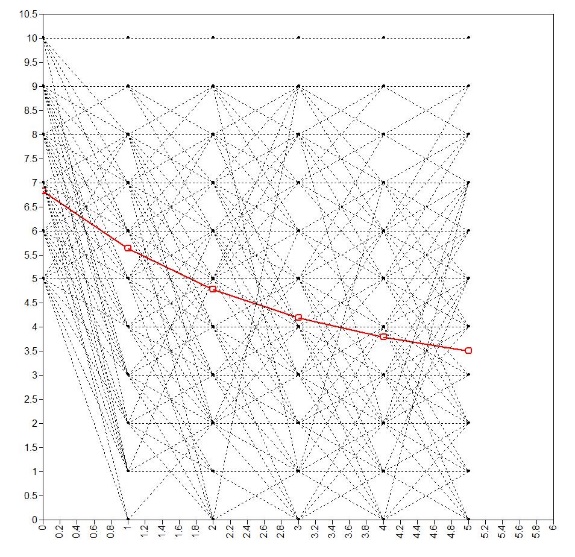
**

Pain score

Pain score

Pain score

Pain score

0 1 2 3 4 5

Visit day

0 1 2 3 4 5

Visit day

0 1 2 3 4 5

Visit day

0 1 2 3 4 5

Visit day

**5-group model 6-group model 7-group model**

**
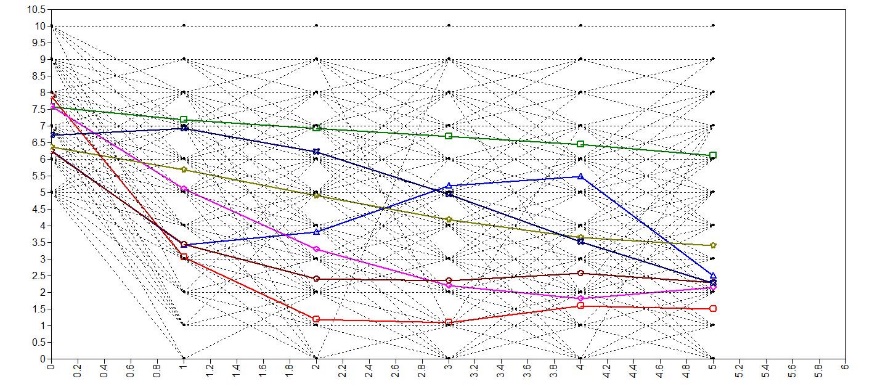

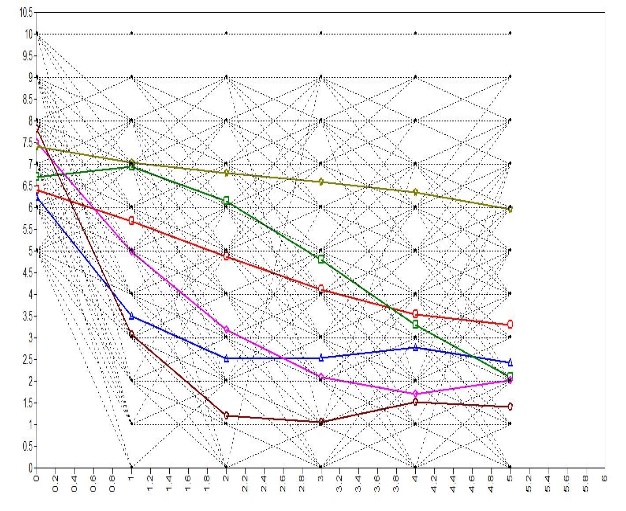

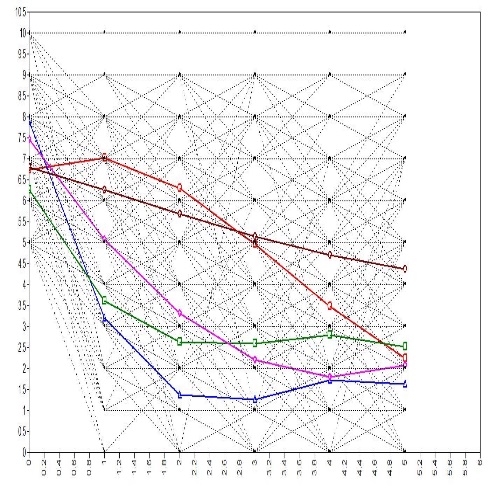
**

Pain score

Pain score

Pain score

0 1 2 3 4 5

Visit day

0 1 2 3 4 5

Visit day

0 1 2 3 4 5

Visit day

**Supplementary Figure 3** Pain score by group-based trajectory membership, diagnosed osteoarthritis only (n=187)

**
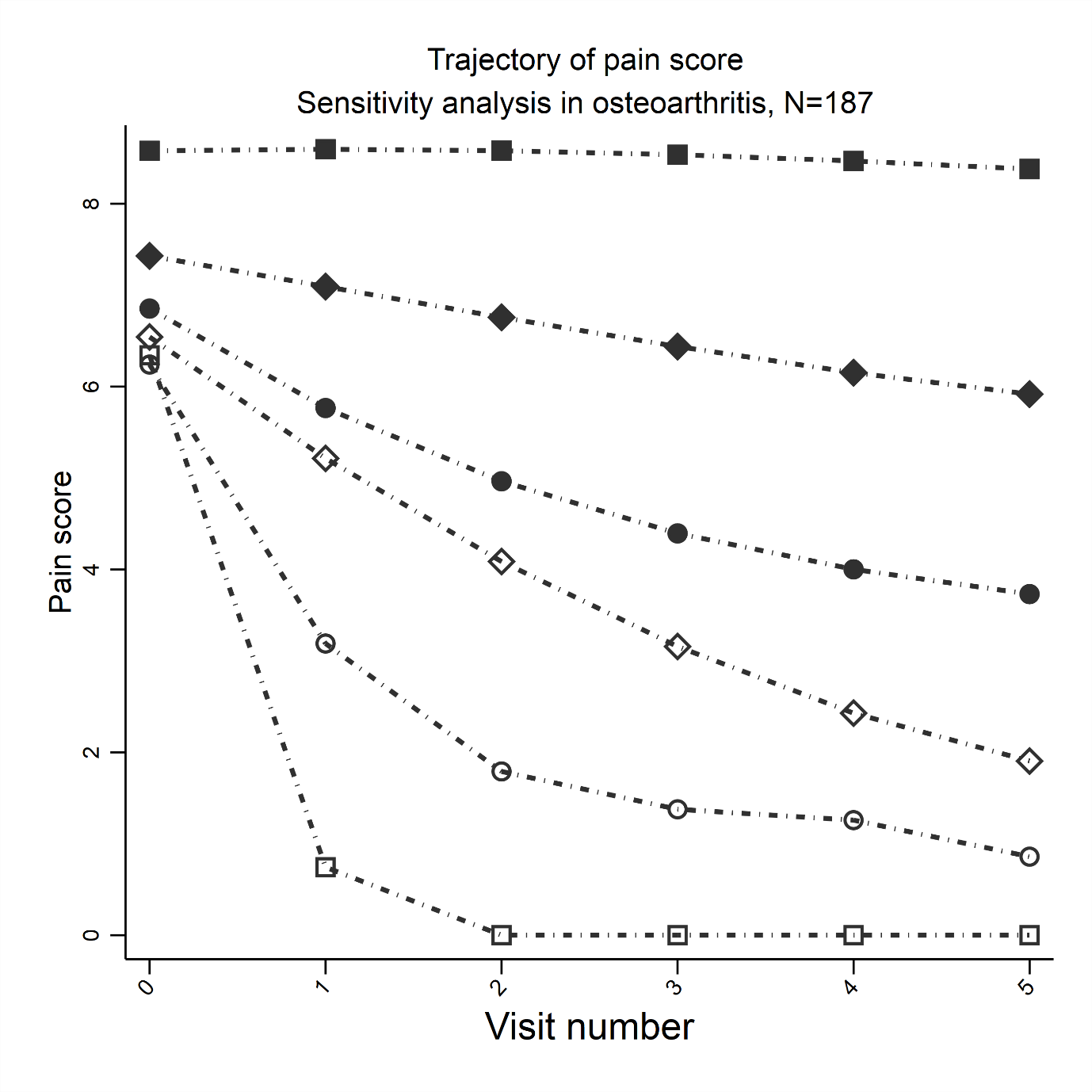
**

On the x-axis, 0 = baseline (no medication), and day one is first treatment day.

Group 1 (n=3) **🞏**

Group 2 (n=27) **🞅**

Group 3 (n=50) **◇**

Group 4 (n=59) ●

Group 5 (n=37) **◆**

Group 6 (n=11) **■**

**Supplementary Figure 4** Pain interference with participant-nominated activity (◼), by pain (⭘) trajectory group, all participants

**
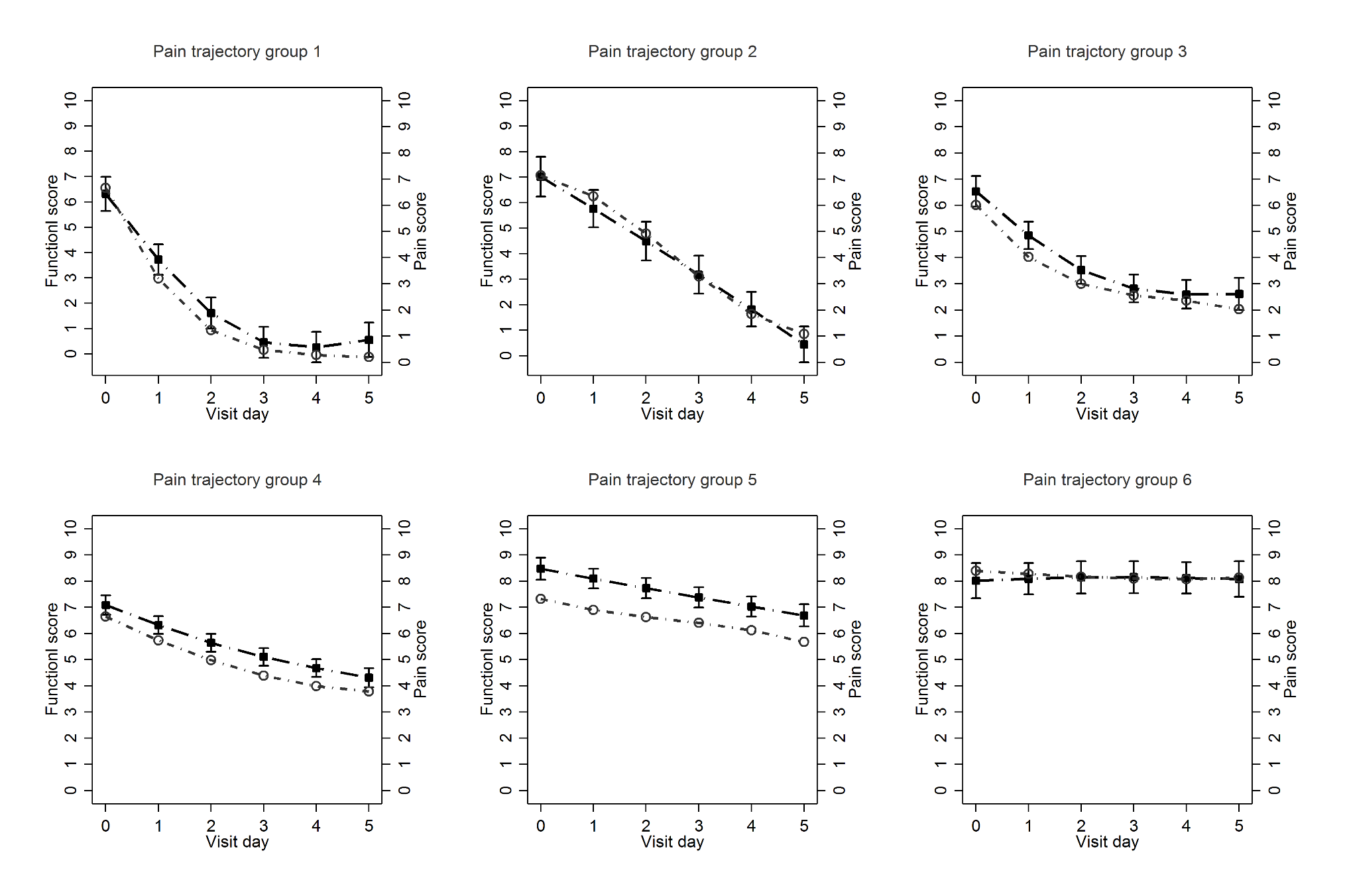
**

**Supplementary Figure 5** Stiffness (◼), by pain (⭘) trajectory group**,** all participants **
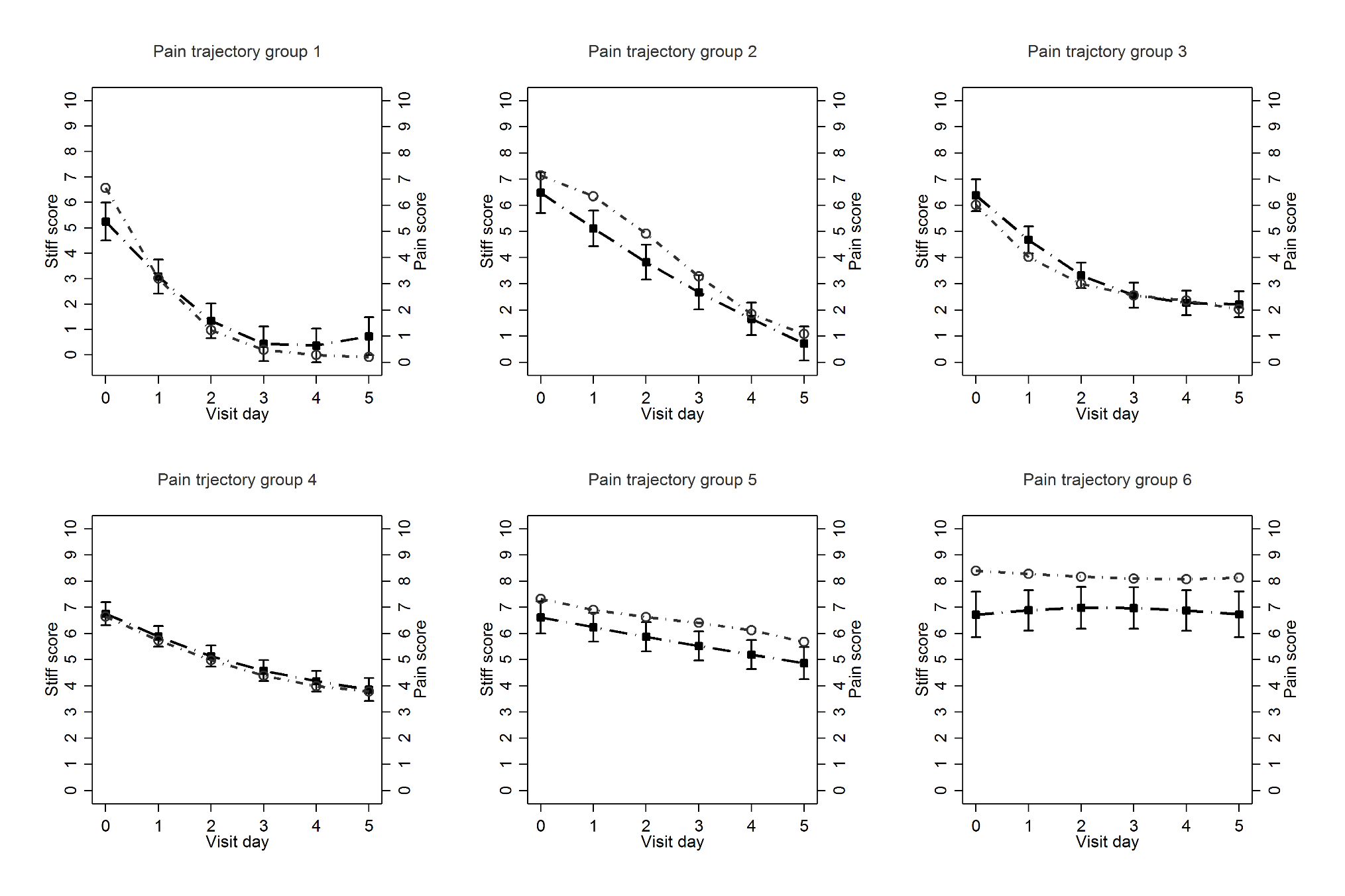
**

**Supplementary Figure 6** Swelling (◼), by pain (⭘) trajectory group, all participants

**
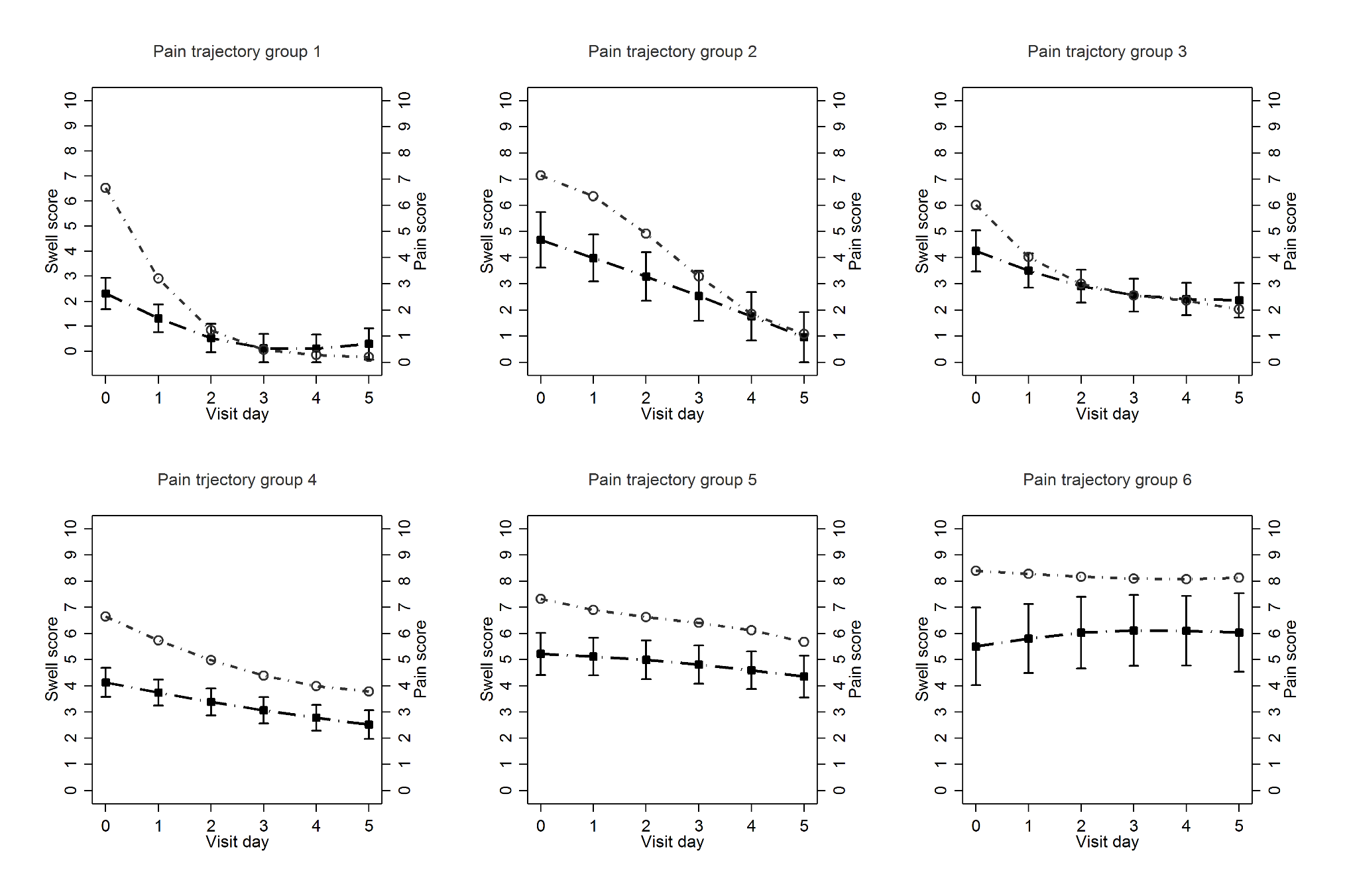
**
